# Live-cell visualization of excitation energy dynamics in chloroplast thylakoid structures

**DOI:** 10.1038/srep29940

**Published:** 2016-07-15

**Authors:** Masakazu Iwai, Makio Yokono, Kazuo Kurokawa, Akira Ichihara, Akihiko Nakano

**Affiliations:** 1 Live Cell Super-Resolution Imaging Research Team, Extreme Photonics Research Group, RIKEN Center for Advanced Photonics, 2-1 Hirosawa, Wako, Saitama 351-0198, Japan; 2 PRESTO, Japan Science and Technology Agency (JST), Honcho, Kawaguchi, Saitama 332-0012 Japan; 3 Institute of Low Temperature Science, Hokkaido University, Sapporo, Hokkaido 060-0819 Japan; 4 Department of Biological Sciences, Graduate School of Science, The University of Tokyo, Bunkyo-ku, Tokyo 113-0033, Japan

## Abstract

The intricate molecular processes underlying photosynthesis have long been studied using various analytic approaches. However, the three-dimensional (3D) dynamics of such photosynthetic processes remain unexplored due to technological limitations related to investigating intraorganellar mechanisms *in vivo*. By developing a system for high-speed 3D laser scanning confocal microscopy combined with high-sensitivity multiple-channel detection, we visualized excitation energy dynamics in thylakoid structures within chloroplasts of live *Physcomitrella patens* cells. Two distinct thylakoid structures in the chloroplast, namely the grana and stroma lamellae, were visualized three-dimensionally in live cells. The simultaneous detection of the shorter (than ~670 nm) and longer (than ~680 nm) wavelength regions of chlorophyll (Chl) fluorescence reveals different spatial characteristics—irregular and vertical structures, respectively. Spectroscopic analyses showed that the shorter and longer wavelength regions of Chl fluorescence are affected more by free light-harvesting antenna proteins and photosystem II supercomplexes, respectively. The high-speed 3D time-lapse imaging of the shorter and longer wavelength regions also reveals different structural dynamics—rapid and slow movements within 1.5 seconds, respectively. Such structural dynamics of the two wavelength regions of Chl fluorescence would indicate excitation energy dynamics between light-harvesting antenna proteins and photosystems, reflecting the energetically active nature of photosynthetic proteins in thylakoid membranes.

Chloroplasts in algae and plants are fundamental organelles inside which water and carbon dioxide are converted into oxygen and carbohydrates through photosynthesis. The initial process in photosynthesis is the capture of light energy, primarily by light-harvesting complex (LHC) proteins in chloroplast thylakoid membranes^1^. The absorbed light energy is rapidly transferred to reaction centers of photosystem I and II (PSI and PSII), where excitation energy is consumed to generate reducing equivalents through photosynthetic electron transport, which results in oxidation of water, reduces NADP^+^, and drives ATP synthesis^2^. Although light energy is essential for photosynthesis, excess excitation energy potentially causes light-induced damage by producing reactive oxygen species. Therefore, photosynthetic machinery in chloroplasts must manage the flow of excitation energy^3^,^4^.

Inside a chloroplast are intricate thylakoid architectures consisting of two distinct membrane regions: the stacked membrane regions (grana) and the stroma-exposed, single-layer membrane regions (stroma lamellae)^5^,^6^. Thylakoid structures have been studied using cryo-electron tomography, revealing the detailed three-dimensional (3D) architecture of grana and stroma lamellae^7^,^8^,^9^. The grana are composed of cylindrical stacks of membranes, and stroma lamellae are unstacked membranes extending from the grana stacks. The impetus for the distinct architectures of these membrane structures is unclear but appears to be related to the localization of the two photosystems: PSI is predominantly located in stroma lamellae, while PSII is usually localized in the grana^10^,^11^,^12^. Intriguingly, the lateral heterogeneity of the two photosystems in thylakoid structures also appears to be related to light-harvesting regulatory mechanisms. In the grana, PSII and LHCII form protein super/megacomplexes arranged in semicrystalline arrays^10^,^13^,^14^, whose organizations are largely affected by light conditions and the composition of LHC proteins^15^,^16^,^17^,^18^,^19^. It has been suggested that the degree of PSII ordered array in grana affects the photosynthetic functions, such as energy transfer, electron transport, photoprotection, and protein repair processes^20^,^21^,^22^,^23^,^24^,^25^. It has also been suggested that the spatial segregation of PSI in stroma lamellae and PSII in grana prevents spillover of excitation energy from PSII to PSI and increases the rate of linear electron transport^26^,^27^,^28^. As the plastoquinone pool becomes highly reduced by unbalanced excitation energy at PSII and PSI, LHCII phosphorylation occurs^29^, inducing phosphorylated LHCII to dissociate from PSII in the grana^30^,^31^, which leads to an increase in the rate of energy transfer to PSI in the stroma lamellae^32^,^33^, presumably via migration of phosphorylated LHCII from the grana to stroma lamellae^34^,^35^. It has been shown that LHCII phosphorylation also causes the formation of PSI-PSII megacomplex, which allows the energy spillover from PSII to PSI in the grana margin^36^,^37^. In addition, during the process of PSII photoinhibition^38^, the damaged PSII is thought to migrate from the grana to grana margins and/or stroma lamellae, where disassembly/reassembly of PSII occurs^39^,^40^. Therefore, the lateral heterogeneity of the two photosystems could be attributed to functional consequences of events that take place in the two different membrane regions: more static conditions in the grana and more dynamic conditions in the stroma lamellae, where protein reorganization occurs.

Previously, we observed thylakoid membrane structural dynamics using a live-cell imaging technique. When using conventional confocal microscopy, we induced macrochloroplast formation in protonemal cells of the moss *Physcomitrella patens* to observe the internal structure clearly. Our live-cell imaging suggested that grana stabilize the overall membrane network structure, but stroma lamellae have structural flexibility^41^. However, there were two limitations preventing us from fully uncovering the dynamics of thylakoid membranes using conventional confocal microscopy. First, due to the low spatial resolution achieved using only a few xy-slices (5 xy-slices with 0.2 μm z-intervals), clear 3D images of thylakoid structures could not be obtained. Second, the low temporal resolution (7 s intervals per voxel) did not provide an adequate view of the continuum of structural changes occurring in both the grana and stroma lamellae. Thus, higher 3D spatiotemporal resolution was needed to precisely visualize entire thylakoid structures and to reveal their structural dynamics.

To achieve live-cell visualization of 3D structural dynamics with spatial resolution beyond the diffraction limit at video rate, we developed super-resolution confocal live imaging microscopy (SCLIM)^42^. SCLIM improves image acquisition of chloroplasts in three ways. First, using an optical instrument with higher magnification (×267 magnification) combined with a high-sensitivity camera device (thousands-fold signal amplification), we can observe thylakoid structures in a chloroplast without inducing macrochloroplast formation. Second, using a spinning-disk confocal scanning unit, we can acquire an xy-image at high speed (30 frames per second). Third, using a piezo actuator at a high repetition rate (z-axis movement ~100 μm at 10–30 Hz), we can obtain a 3D image of a whole chloroplast within seconds. In addition, 3D deconvolution greatly increases spatial resolution by accurately determining photon emitting localizations through reverse calculation of the convolved point-spread function^43^. SCLIM has already been successfully applied to visualize the dynamics of membrane trafficking in eukaryotic cells^44^,^45^,^46^,^47^.

Here, in an effort to uncover what happens inside a chloroplast, we used SCLIM to examine dynamic aspects of the entire thylakoid architecture in live *P. patens* protonemal cell, which is an excellent imaging model system to investigate cell biology in live cells. We successfully used SCLIM to differentiate between grana and stroma lamellae within chloroplasts in live cells. SCLIM also revealed the spatial heterogeneity of chlorophyll (Chl) fluorescence emission by detecting two different wavelength regions (shorter than ~670 nm and longer than ~680 nm) simultaneously. By measuring Chl fluorescence emission using grana membrane fractions, we determined that free LHCII had more contributions to the shorter wavelength region than the longer one. The high-speed 3D time-lapse imaging also revealed a different rate of dynamics of the two different wavelength regions, which might be related to the local Chl-binding protein reorganization in thylakoid membranes. Our work demonstrates that the high-speed 3D time-lapse imaging with spatial resolution beyond the diffraction limit in live-cell system will be an important analytic approach to explore the complicated but fascinating photosynthetic mechanisms in chloroplasts.

## Results and Discussion

### Chl fluorescence observed by SCLIM reveals the internal architecture of a chloroplast in live *P. patens* cells

**Figure 1:**
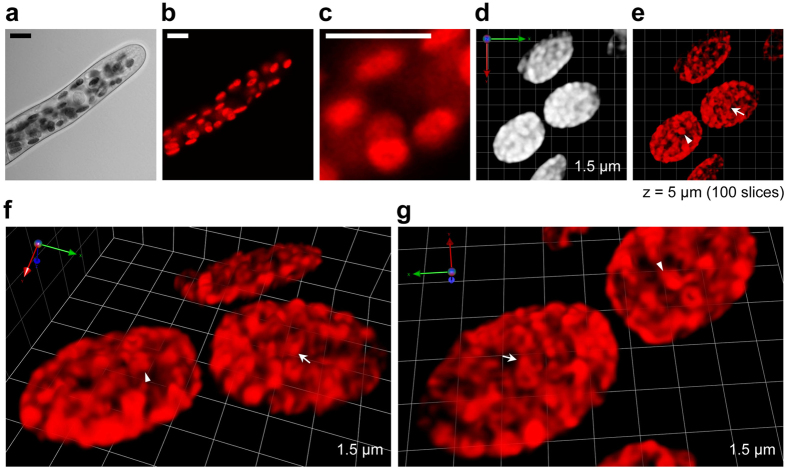
SCLIM observation of chloroplasts in a live WT *P. patens* protonemal cell. Chloroplasts in *P. patens* protonema grown on agar medium in a glass-bottom dish were observed directly. (**a**–**c**) 3D images of WT *P. patens* chloroplasts observed using conventional confocal microscopy. (**a**) Differential interference contrast image, (**b**) Chl fluorescence image, and (**c**) Chl fluorescence image with the same scale as in (**d,e**). The 3D image was taken using five optical sections (x, y) sequentially acquired at 0.2 μm intervals (z = 1 μm) as previously described^41^. (**d**–**g**) 3D images of WT *P. patens* chloroplasts observed using SCLIM. (**d**) Chl fluorescence image before 3D deconvolution, (**e**) Chl fluorescence image after 3D deconvolution, and (**f**,**g**) close-up views of (**e**) from different angles. Arrows and arrowheads indicate the same structures in different figures (**e**–**g**). 3D image reconstructed using 100 optical sections (x, y) sequentially acquired at 0.05 μm intervals (z = 5 μm). Scale bars are 10 μm (**a**–**c**). The length indicates the scale of each unit (**d**,**f**,**g**). Hereafter, all images were reconstructed with 3D deconvolution. See also Supplementary Movie 1.

**Figure 2:**
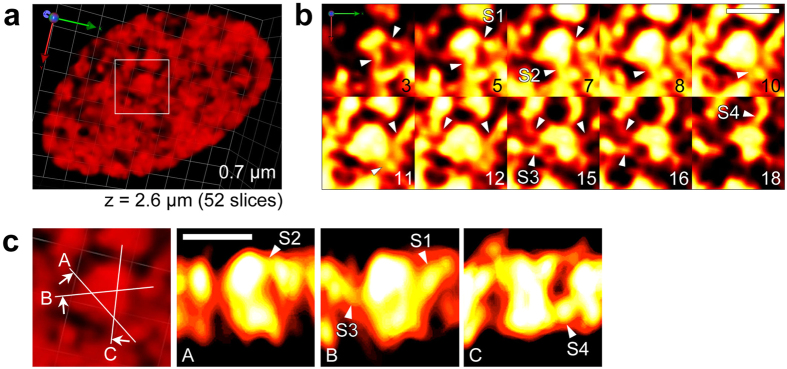
3D architectures within a chloroplast in a live WT *P. patens* protonemal cell. (**a**) 3D image of WT *P. patens* chloroplasts observed using SCLIM. The 3D image was reconstructed using 52 optical sections (x, y) sequentially acquired at 0.05 μm intervals (z = 2.6 μm). (**b**) Optical sections of a granum (shown in the square in (**a**)) from top to bottom. Number indicates slice numbers. Arrowheads indicate apparent stroma lamellae structures extending from grana margins at different positions. (**c**) Side views of the same granum structure (square in (**a**)) from different angles of the vertical cross section (A–C). Arrowheads labeled S1–S4 indicate the same structures as those in (**b**). The length indicates the scale of each unit. Scale bars are 1 μm (**b**,**c**).

We observed Chl fluorescence emitted from normal-sized chloroplasts in live *P. patens* protonemal cells, as shown previously^41^. It is often difficult to observe distinct structures inside each chloroplast using conventional confocal microscopy (Fig. 1a–c). However, SCLIM overcomes this problem, as described above. Using 100 xy-slices at 0.05-μm z-intervals (total length of z-axis = 5 μm), we obtained single 3D images covering entire chloroplasts in live cells (Fig. 1d). After 3D deconvolution, the whole thylakoid structure in the chloroplasts could be visualized, and distinct disk-like structures, or grana, were clearly observed (Fig. 1e–g; Supplementary Movie 1). Around a granum, several thread-like structures, or stroma lamellae, were also visible (Fig. 2a). Previously, we could not observe the side-view of a granum due to the low axial resolution obtained using only a few z-slices^41^. Here, SCLIM revealed the side-view of a granum and showed that the stroma lamella extended from parts of grana margins at different heights (Fig. 2b,c). Although the spatial resolution achieved by SCLIM is still far from comparable to the one obtained by electron microscopy, SCLIM achieves to visualize the overall Chl fluorescence structures within a chloroplast three-dimensionally in live cells.

### GFP analysis verifies the Chl fluorescence structures revealed by SCLIM

**Figure 3:**
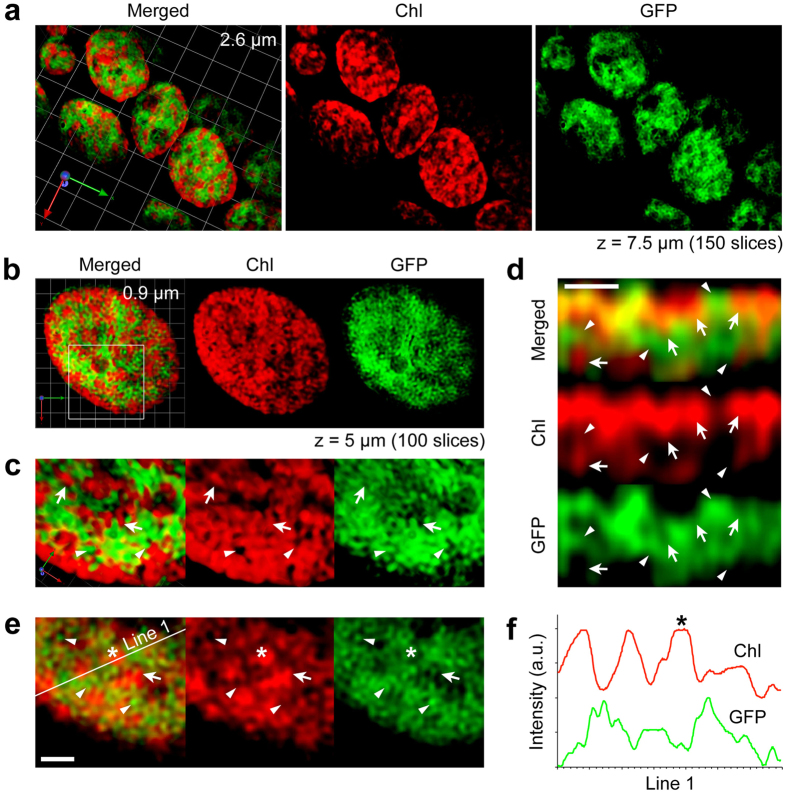
Verification of structures observed using SCLIM and reconstructed by 3D deconvolution. *P. patens* line in which GFP is overexpressed in the stroma, as observed using SCLIM. (**a**) Chloroplasts observed in live cells at two different wavelength regions for Chl and GFP. (**b**) Close-up view of a chloroplast. (**c**) More magnified view of the same chloroplast as in (**b**) from a different angle. (**d**) Composite 2D image (yz) of the same chloroplast. (**e**) Composite 2D image (xy) of the same chloroplast (square in (**b**)). Arrows and arrowheads indicate the structures for Chl and GFP, respectively. (**f**) Line scan of fluorescence intensity in ‘Line 1’ (shown in (**e**)). Asterisk corresponds to the position shown in (**e**). The length indicates the scale of each unit (**a**,**b**). Scale bars are 1 μm (**d**,**e**). The number of optical sections (x, y) and the depth (z) of 3D image are shown (**a**,**b**). See also Supplementary Movie 2.

Because this work represents unprecedented live-cell visualization of whole 3D Chl fluorescence structures inside a chloroplast, we evaluated whether such structures are physiologically relevant. We therefore generated a *P. patens* line in which green fluorescent protein (GFP) was overexpressed in the chloroplast stroma. If our 3D imaging analysis functions properly, Chl and GFP fluorescence should be present at different positions, as the former should be in thylakoid structures whereas the latter should be in the chloroplast stroma. Indeed, most Chl and GFP fluorescence signals did not overlap (Fig. 3a; Supplementary Movie 2). The 3D image revealed thin structures for both fluorescence signals, but even those were separate from each other (Fig. 3b,c). Images created from two-dimensional (2D) slices of side-viewed chloroplasts demonstrated that the structures revealed by Chl and GFP fluorescence were spatially excluded from each other (Fig. 3d–f). Thus, we conclude that SCLIM reveals *bona fide* thylakoid structures in chloroplasts within a live cell. It should be emphasized, however, that we observed Chl fluorescence, not actual thylakoid lipids, suggesting that the structural differences should exist according to the methodological differences between electron microscopy and fluorescence microscopy.

### Different wavelength regions of Chl fluorescence emission reveal different spatial characteristics within thylakoid structures

**Figure 4:**
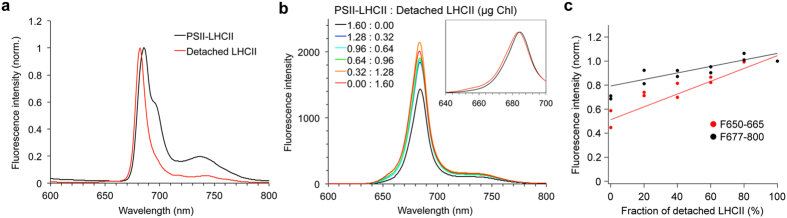
Spectroscopic analyses of two different wavelength regions of Chl fluorescence. (**a**) 77 K fluorescence spectra using grana membranes with or without a detergent (Triton X-100) treatment. Grana membranes without a detergent treatment contain LHCII associated with PSII, forming PSII-LHCII supercomplexes in the membranes (black line). The detergent treatment solubilized PSII-LHCII supercomplex, causing LHCII detached from PSII (red line). Chl concentration of both samples was adjusted to the same (1.6 μg Chl/mL). (**b**) Room-temperature fluorescence spectra using the mixture of PSII-LHCII supercomplex and detached LHCII at a different ratio. The Chl amount of each sample was adjusted to the same (1.6 μg Chl). The ratio shown in legend indicates the Chl amount (μg) of PSII-LHCII and detached LHCII included in each sample. The inset shows the room-temperature fluorescence spectra of PSII-LHCII supercomplex (grana membranes) and detached LHCII (grana membranes treated with a detergent) normalized at the peak. (**c**) The values of Chl fluorescence intensity measured in the two different wavelength regions (F650-665 and F677-800), which were the same as the filter properties used in SCLIM observation, were plotted against the fraction of detached LHCII in the sample. The graph was normalized to the value obtained in the sample with 100% detached LHCII. Excitation wavelength was 473 nm, which is the same as we used in SCLIM observation.

Chl fluorescence emission at room temperature is observed from ~645 to ~760 nm, with a major peak at around 680 nm, which is considered to originate from PSII as a terminal energy acceptor^1^. Although PSI also functions as a terminal energy acceptor, its fluorescence emission is barely detectable at room temperature^48^ (Supplementary Fig. 1). Thus, most Chl fluorescence emission observed under our SCLIM conditions likely reflects the localization of PSII. Although SCLIM is not capable of spectral imaging analysis, two optical filters are applicable to detect two different wavelength regions of Chl fluorescence shorter than ~670 nm and longer than ~680 nm. To characterize these two wavelength regions, we first analyzed isolated grana membranes by fluorescence spectroscopy. Low-temperature (77 K) fluorescence spectra of grana membranes showed a peak at 686 nm with a shoulder at 695 nm, which indicates that energy transfer from LHCII to PSII core antenna (CP43 and CP47) occurs (Fig. 4a, PSII-LHCII). By solubilizing grana membranes with a detergent (Triton X-100), the peak was shifted shorter to 682 nm, and the shoulder at 695 nm decreased significantly, suggesting that most LHCII is detached from PSII core (Fig. 4a, Detached LHCII). We next compared Chl fluorescence intensity at room-temperature using the mixture of solubilized and unsolubilized grana membranes. The results indicate that higher Chl fluorescence intensity was observed when more detached LHCII was included in the sample (Fig. 4b). Although the peak of Chl fluorescence at room temperature becomes broader as compared to the one observed at 77 K, the effect of detached LHCII was still observable at room temperature as the peak was shifted from 685 nm to 683.5 nm (Fig. 4b, inset). To observe how detached LHCII would affect the Chl fluorescence observation by SCLIM, we analyzed the change in fluorescence intensity of the two different wavelength regions that SCLIM can detect separately. The results indicated that the shorter wavelength region was twice more sensitive to the detection of detached LHCII than the longer one (Fig. 4c). Because it is almost impossible to unambiguously identify each Chl-binding protein using current live-cell imaging technique without detecting GFP-tagged proteins or fluorescence lifetime of fluorescent molecules at the single-molecule level, we cannot ascertain which detached LHCIIs are involved specifically in the shorter wavelength region in this study. Therefore, we hereafter consider that Chl fluorescence observed in the shorter and longer wavelength regions has more influence on antenna pigment molecules (APMs) and PSII, respectively.

**Figure 5:**
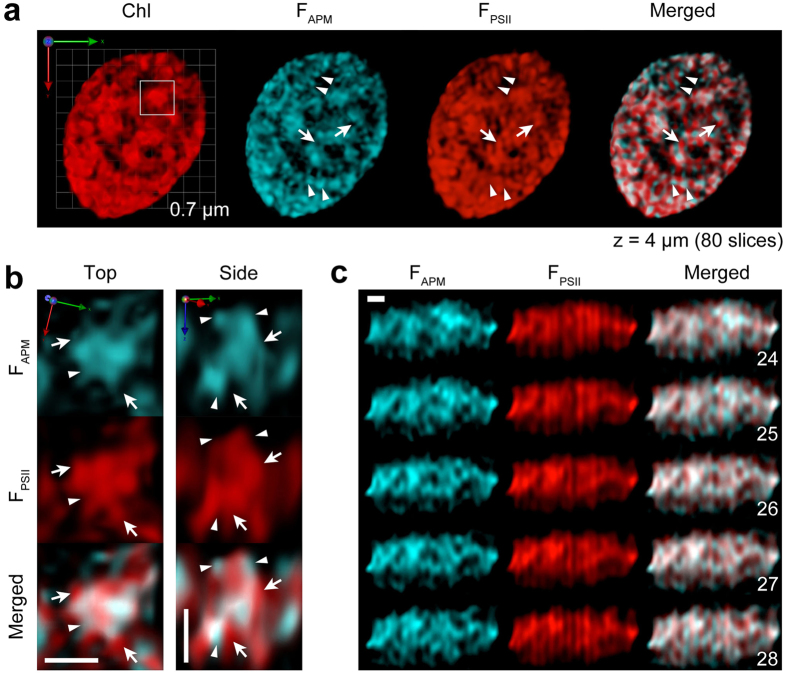
Different spatial characteristics and different energy states of Chl fluorescence in thylakoid structures. (**a**) SCLIM observation of a chloroplast in a live WT *P. patens* protonemal cell with two different wavelength regions shorter and longer than 680 nm (F_APM_ and F_PSII_, respectively). The length indicates the scale of each unit. (**b**) Close-up views (top and side) of a granum (square in (**a**)) observing two different wavelength regions. Arrows and arrowheads indicate the structures for F_APM_ and F_PSII_, respectively. (**c**) Optical sections (yz) of a chloroplast observed at two different wavelength regions. Numbers indicate slice numbers. Scale bars are 0.5 μm (**b**,**c**). The number of optical sections (x, y) and the depth (z) of 3D image are shown (**a**). See also Supplementary Movie 3.

Due to the high-sensitivity camera detection system of SCLIM, Chl fluorescence wavelengths shorter than ~670 nm (putatively representing APMs) were detectable, and the 3D image shows the structural proximity of these signals to those from wavelengths longer than ~680 nm (putatively representing PSII) (Fig. 5a). Interestingly, however, a certain number of structures emitting fluorescence shorter than ~670 nm did not overlap with those emitting wavelengths longer than ~680 nm. Even within a granum, the localizations of both fluorescence signals were different (Fig. 5b). This finding indicates that these two fluorescence regions reflect two different populations with different energy states. As energy transfer normally occurs from shorter to longer wavelengths (*i.e*., from an energy donor to an energy acceptor), fluorescence signals shorter than ~670 nm and longer than ~680 nm most likely reflect any APM- and PSII-populated regions (F_APM_ and F_PSII_, respectively). In fact, in images of the 2D xz-slices of the side-viewed chloroplast, fluorescence signals longer than ~680 nm revealed several vertical structures, which most likely reflect stacked grana structures, where PSII is mainly located (Fig. 5c, F_PSII_). Some F_APM_ signals reflected structures crossing the F_PSII_ vertical structures (Fig. 5c, F_APM_; Supplementary Movie 3). As mentioned earlier, PSI fluorescence is barely observable and therefore, F_APM_ reflects a population of APMs in thylakoid membranes that is not transferred to any photosystems. Interestingly, F_APM_ revealed rather irregular structures compared to the distinct vertical structures of F_PSII_, which indicates that the localization of APMs is not restricted to grana. Also, due to the distinctiveness of F_PSII_ vertical structures, it is easily noticeable that F_APM_ showed a bridge-like structure between F_PSII_ vertical structures. Such structures of F_APM_ most likely represent stroma lamellae, where PSI is predominantly located. Interestingly, it has been shown that the stroma lamellae in *P. patens* contain more LHCII proteins as compared to the ones in *Arabidopsis thaliana*^49^. Therefore, together with our observation that the contribution of LHCII fluorescence to F_APM_ is more than that to F_PSII_ (Fig. 4), SCLIM could be successfully used to visualize side views of grana-like and stroma lamellae-like structures in live cells by differentiating the two different wavelength regions of Chl fluorescence.

It should be mentioned that the axial resolution of a microscope optical system is generally approximately two-times lower than the lateral resolution, and therefore we cannot exclude the possibility that the side-view image is slightly stretched along the z-axis. However, the SCLIM system scans each xy-slice image with a z-interval of 0.05 μm to capture sufficient signals to improve the axial resolution of images reconstructed by 3D deconvolution and can therefore reveal the different structures of F_APM_ and F_PSII_.

The high-sensitivity camera detection of the SCLIM system thus successfully revealed the localization of F_APM_, whose intensity was lower than that of F_PSII_ (we estimate that the intensity of F_APM_ was approximately 12% that of F_PSII_ in a bulk observation). We confirmed that F_APM_ detected by SCLIM was not an enhanced signal from noise (Supplementary Figs 2 and 3). F_APM_ and F_PSII_ were detected using two different cameras that were optimized independently for detection of two different regions of fluorescence emission. Accordingly, the images must be interpreted based on the localization, rather than the intensity of signal for each fluorophore. This also implies that we cannot quantify accurately whether the signal indicates Fo or Fm as determined by PAM fluorometry based on different principles and techniques. Therefore, what we can deduce from the fluorescence imaging microscopy analysis herein is the spatial relationships between fluorophores. Because fluorophores emit fluorescence with only one energy level (wavelength) at a time, our observations of various populations of F_APM_ and F_PSII_ in different locations at the same time suggest that several APMs, presumably free LHCII proteins, exist in individual domains separately from any photosystem, as suggested previously^50^.

### 3D time-lapse imaging reveals excitation energy dynamics in thylakoid structures in live plant cells

**Figure 6:**
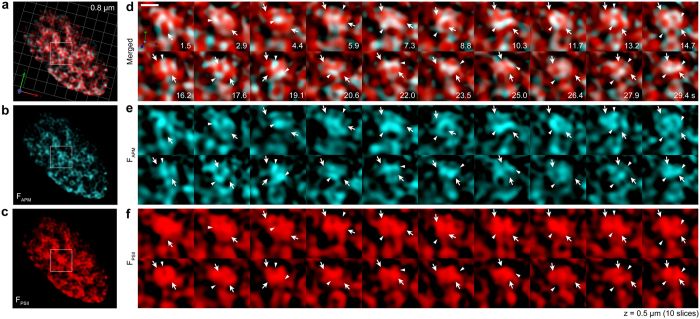
Excitation energy dynamics revealed by 3D time-lapse imaging. A chloroplast in a live WT *P. patens* protonemal cell observed using SCLIM. 3D time-lapse imaging was performed by acquiring the 3D image (xy with 10 optical sections with 0.05-μm z-intervals; z = 5 μm) sequentially (t = ~1.4 s/each 3D image) with observation at F_APM_ and F_PSII_ regions simultaneously. (**a**–**c**) Overview of a chloroplast. The length indicates the scale of each unit. (**d**–**f**) 3D time-lapse imaging of a granum (square in (**a**–**c**), respectively). Arrows and arrowheads indicate the structures for F_PSII_ and F_APM_, respectively. Scale bar is 0.5 μm (**d**). See also Supplementary Fig. 5 and Movie 4.

Next, we performed 3D time-lapse imaging analysis of single chloroplasts in live cells. The results indicate that the F_APM_ showed more dynamic aspects than the comparatively stationary F_PSII_ (Fig. 6). The overall position of F_PSII_ was mostly unchanged, as it reflects a granum, but the outlined shape was varying with a slight oscillatory motion (Fig. 6f). On the other hand, the position and shape of F_APM_ dynamically changed, but the actual movement could not be traced explicitly because this random motion occurred at a rate faster than ~1.5 s (Fig. 6e). As described for Fig. 5, some of the positions of F_APM_ and F_PSII_ did not overlap (Fig. 6d–f, arrows and arrowheads; Supplementary Movie 4), suggesting that the changes in the positions of both F_APM_ and F_PSII_ might be correlated. Because F_APM_ reflects the APM-populated regions, from which excitation energy is transferred to PSII, the dynamics in the position and shape of F_APM_ observed within ~1.5 s are greatly affected by changes in the excitation levels at each PSII reaction center. Thus, the observed dynamics of F_APM_ revealed by SCLIM reflect the excitation energy dynamics that occur within thylakoid structures in live cells.

It has been known that the various types of PSII-LHCII supercomplexes existed in grana, suggesting that various types of energy transfer with different fluorescence lifetime could occur depending on the organization and connectivity of PSII and LHCII^51^. The intensity of Chl fluorescence emission from LHCII is also shown to be varied depending on its antenna size^52^. Therefore, our observation might visualize the dynamic change in the rate of energy transfer between APMs and PSII due to (1) fluctuations in the saturation of excitation energy at PSII reaction centers, which is largely affected by the redox state of plastoquinone pool, (2) a local change in interactions between APMs and PSII that modulates the rate of energy transfer between them, and/or (3) a local change in the antenna size of APMs. Another possible factor in F_APM_ dynamics is the effect of the energy transfer at grana margins and stroma lamellae, where excitation energy state of PSII and PSI is largely variable depending on the antenna size^53^. Because PSI fluorescence is very limited at room temperature, the transient interaction between APMs and PSI could cause the random fluctuation of F_APM_. Also, a recently described PSI-PSII megacomplex in which quenching of excitation energy at PSI occurs could contribute to F_APM_ dynamics^36^. Together our observations demonstrate that the high sensitivity and high spatiotemporal resolution of SCLIM allowed us to visualize excitation energy dynamics, which could not be observed previously^41^.

Our simultaneous observation of two different wavelength regions of Chl fluorescence (F_APM_ and F_PSII_) implies that the nature of thylakoid membranes in live cells is energetically dynamic in a three-dimensional manner. According to the spatial characterization in the tens- to hundreds-nanometer scale revealed by SCLIM, excitation energy dynamics within a thylakoid structure vary considerably within 1.5 seconds, although we cannot be certain that such dynamics also reflect actual structural modifications of the membranes as we observed fluorescence. Thus, we do not provide the evidence for the relationship between the excitation energy dynamics and the actual structural membrane dynamics. Given the fact that thylakoid membranes are filled with Chl-binding proteins, the spatial dynamics observed in a very short period of time would indicate the excitation energy dynamics. The finding that the degree of dynamics between F_APM_ and F_PSII_ varied, exhibiting more dynamic and more static conditions, respectively, suggests that different pigment-binding proteins are involved in these two regions, with F_APM_ and F_PSII_ reflecting stroma lamellae and grana, respectively. Both dynamic and static conditions would be achievable due to the exceptional characteristics of thylakoid membranes, in which photosynthetic proteins are highly crowded but still mobile in the fluid lipid bilayer^5^,^54^,^55^. The excitation energy dynamics of thylakoid membranes observed by SCLIM might therefore reflect the structural stability of grana and the structural flexibility of stroma lamellae, which could be crucial for functional adjustments of the light-harvesting capacity of the photosystems.

## Methods

### Plant materials and growth conditions

WT *P. patens* (Gransden 2004) protonemata were cultured as described previously^56^. The *P. patens* line in which sGFP was overexpressed in the stroma was cultured in a similar manner, except for the addition of Geneticin (Invitrogen) at a final concentration of 20 mg/L.

### Generation of transgenic moss

Using the pPpMADS-Actin plasmid, site-directed mutagenesis was performed by polyethylene glycol-mediated transformation^56^ to integrate the DNA sequences for sGFP^57^, in which the sequence encoding the transit peptide sequence of a ribulose-1,5-bisphosphate carboxylase/oxygenase small subunit of *Arabidopsis thaliana* (*AtRbcS*)^58^ was fused to that for the N-terminal region (with a polyglycine linker) and inserted into the *P. patens MADS2* neutral site (see also Supplementary Fig. 4), resulting in overexpression of sGFP in the stroma.

### Spectroscopic analyses

Grana membranes were prepared from spinach leaves according to the established method^59^. The spectroscopic analyses were performed in the buffer containing 25 mM BisTris/HCl (pH7.0), 20% (w/v) glycerol, and 0.05% (w/v) digitonin, using a U-3310 absorption spectrophotometer (Hitachi) and F-2500 fluorescence spectrophotometer (Hitachi) as described previously^36^. The final concentration of 3% (w/v) Triton X-100 was used to solubilize LHCII completely from grana membranes. The Chl concentration and *a*/*b* ratio were determined as described previously^60^.

### SCLIM

*P. patens* protonemal cells were prepared for observation according to our previous study^41^. The SCLIM setup was described previously^42^,^46^, except that three EM-CCD cameras (Hamamatsu Photonics, Japan) were used to detect GFP, F_APM_, and F_PSII_ fluorescence simultaneously. A solid-state laser with emission at 473 nm (Cobolt Blues) was used to excite both Chl and GFP. Three optical filters (497–535 nm bandpass, 650–665 nm bandpass, and 677 nm longpass) were used to detect GFP, F_APM_, and F_PSII_ fluorescence, respectively. Optical serial z-sections were acquired at 0.05 μm intervals. For 3D time-lapse imaging, 3D images were acquired sequentially without additional interval time. 3D deconvolution analysis was performed in blind mode using Volocity software (Improvision). Random noise signals were eliminated by calculation with software to reduce the uncertainty inherent in measuring weak signals. Extraction of 2D slices from 3D voxels was performed using ImageJ software (US National Institutes of Health).

## Additional Information

**How to cite this article**: Iwai, M. *et al*. Live-cell visualization of excitation energy dynamics in chloroplast thylakoid structures. *Sci. Rep*. **6**, 29940; doi: 10.1038/srep29940 (2016).

## Supplementary Material

Supplementary Information

Supplementary Movie S1

Supplementary Movie S2

Supplementary Movie S3

Supplementary Movie S4
