# Supplementary material for: Live-cell visualization of excitation energy dynamics in chloroplast thylakoid structures
Source: Sci Rep. 2016 Jul 15;6:29940. doi: 10.1038/srep29940 (PMC4945916; doi:10.1038/srep29940)
Supplement: Supplementary Information [file srep29940-s1.pdf]

Supplementary Information

Live-cell visualization of excitation energy dynamics in chloroplast thylakoid structures

Masakazu Iwai, Makio Yokono, Kazuo Kurokawa, Akira Ichihara & Akihiko Nakano

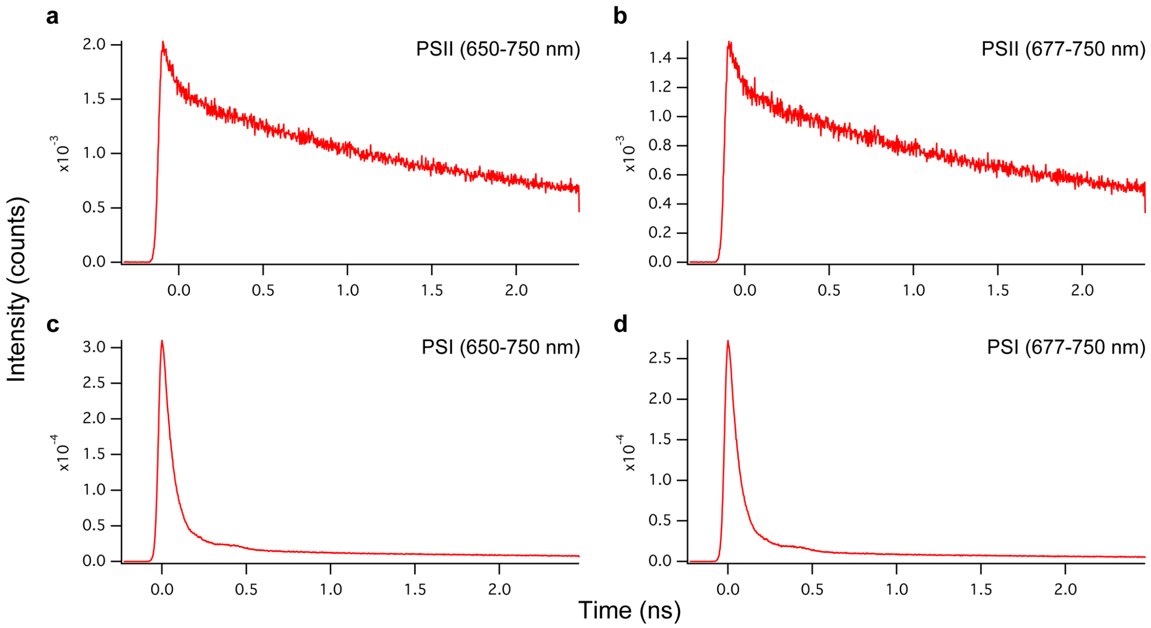

Supplementary Figure 1 | Time-correlated single-photon counting of purified PSI and PSII samples. Both PSI and PSII samples were purified from *Chlamydomonas* according to the previous study (Takahashi et al., PNAS, 2006). TCSPC measurement was performed at room temperature according to the previous study (Yokono et al., *Biochim. Biophys. Acta*, 2008). Fluorescence decay curves of purified PSII observed at (**a**) 650-750 nm and (**b**) 677-750 nm. Fluorescence decay curves of purified PSI observed at (**c**) 650-750 nm and (**d**) 677-750 nm. Each decay curve was normalized by averaged fluorescence lifetime. The data indicate that fluorescence lifetime of PSI is more than 40 times shorter than that of PSII. Even without a bandpass filter to cut PSI fluorescence, the contribution of PSI fluorescence is 2.4% of total photon counts of PSII fluorescence observed at 677-750 nm.

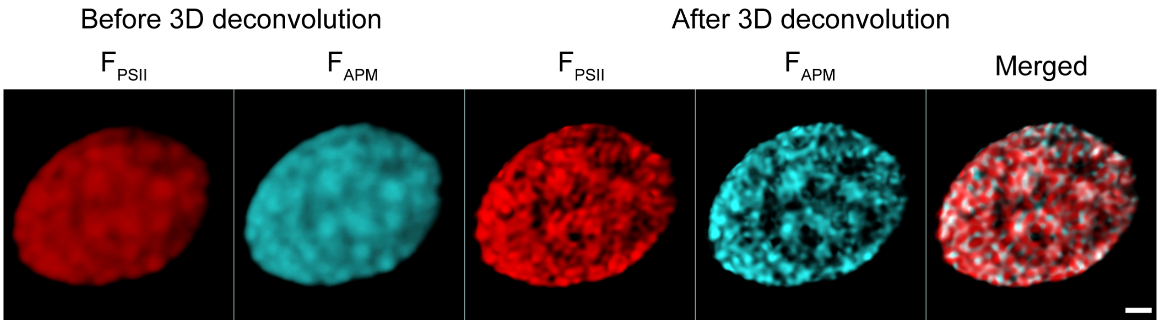

Supplementary Figure 2 | Comparison of the images before and after 3D deconvolution. The 3D image of WT *P. patens* chloroplasts were observed using SCLIM with two different wavelength regions shorter and longer than ~680 nm (F_{APM} and F_{PSII}, respectively). As described in the main text, 3D deconvolution increases spatial resolution of the image observed three-dimensionally. Before 3D deconvolution, the image contained noisy signals from out-of-focus blur. After 3D deconvolution, such signals were reduced, and the photon emitting points were determined through reverse calculation of the convolved point-spread function. Therefore, the 3D images for both F_{PSII} and F_{APM} were reconstructed using the signals which were sufficiently larger than noise. Scale bar, 1 μm.

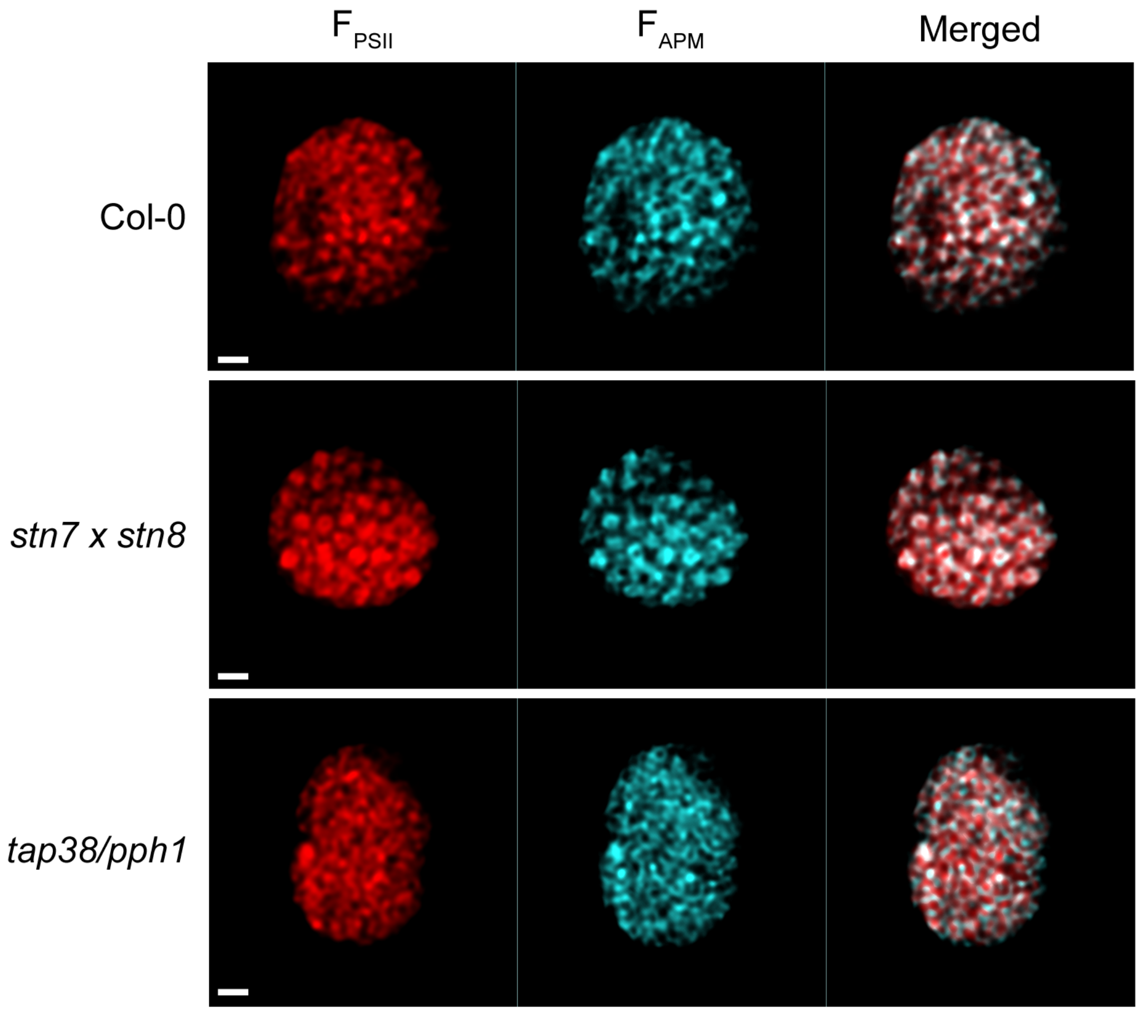

Supplementary Figure 3 | SCLIM observation using the isolated chloroplasts from *Arabidopsis thaliana*. The 3D images of the intact chloroplast isolated from *A. thaliana* WT (Col-0), *stn7* x *stn8*, and *tap38*/*pph1* mutants were observed using SCLIM with two different wavelength regions shorter and longer than ~680 nm (F_{APM} and F_{PSII}, respectively). The results indicated that as compared to the chloroplast from WT, the one from *stn7* × *stn8* mutant showed broader grana, and the one from *tap38*/*pph1* mutant showed smaller grana, both as previously observed in transmission electron microscopy (Fristedt *et al*., *Plant Cell*, 2009; Armbruster *et al.*, *Plant Cell*, 2009). Thus, SCLIM has the ability to visualize the differences in thylakoid structures for both F_{APM} and F_{PSII}. This confirms that the structures revealed through F_{APM} observation were not based on noise. Scale bars = 1 μm.

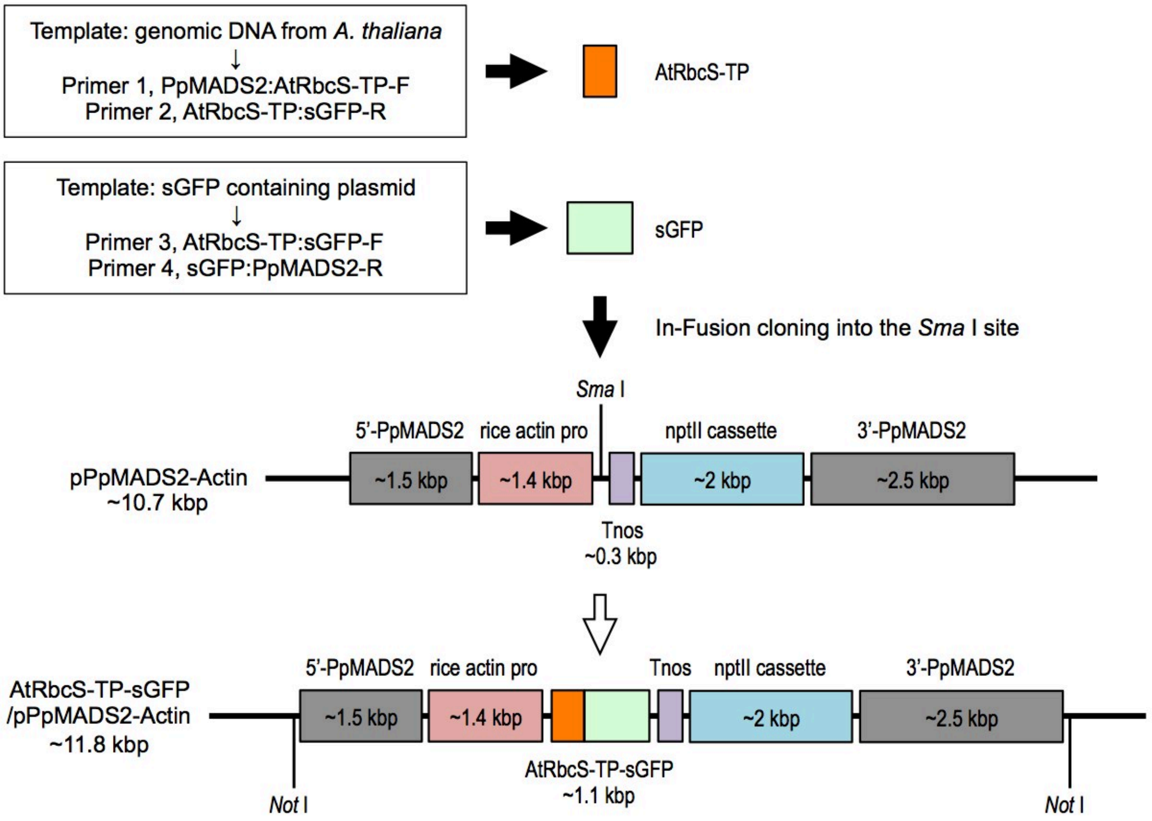

Supplementary Figure 4 | Generation of a *P. patens* line in which GFP is overexpressed in the chloroplast stroma. First, the DNA sequence for the transit peptide of ribulose-1,5-bisphosphate carboxylase/oxygenase small subunit of *Arabidopsis thaliana* (AtRbcS-TP) was amplified by PCR using Primers 1 and 2 with *A. thaliana* genomic DNA as a template. Next, the DNA sequence for sGFP was amplified by PCR using Primers 3 and 4 with any plasmids containing a whole sequence of sGFP. Both ends of the two amplified PCR fragments contained the sequence for In-Fusion Cloning technology (Clontech). Also, the 3' end of amplified AtRbcS-TP contained the sequence for a linker (GGGGVD) between AtRbcS-TP and sGFP. The two PCR fragments were inserted simultaneously into the *Sma* I site in the pPpMADS2-Actin plasmid using In-Fusion Cloning technology (Takara). The generated AtRbcS-TP-sGFP/pPpMADS2-Actin was excised with restriction enzyme *Not* I, and 8.8 kbp of the purified DNA fragment was used for gene targeting by polyethylene glycol-mediated transformation.

Supplementary Figure 5 | The images for the entire chloroplast as shown in Fig 6. The time-lapse images as shown in Fig. 6 in the main text are cropped images as indicated in square in time point #1. The location of chloroplast was not significantly changed during the observation. The cropped images in Fig. 6 indicated the same structure at the same position in the chloroplast. The details for the observation conditions are in the main text.

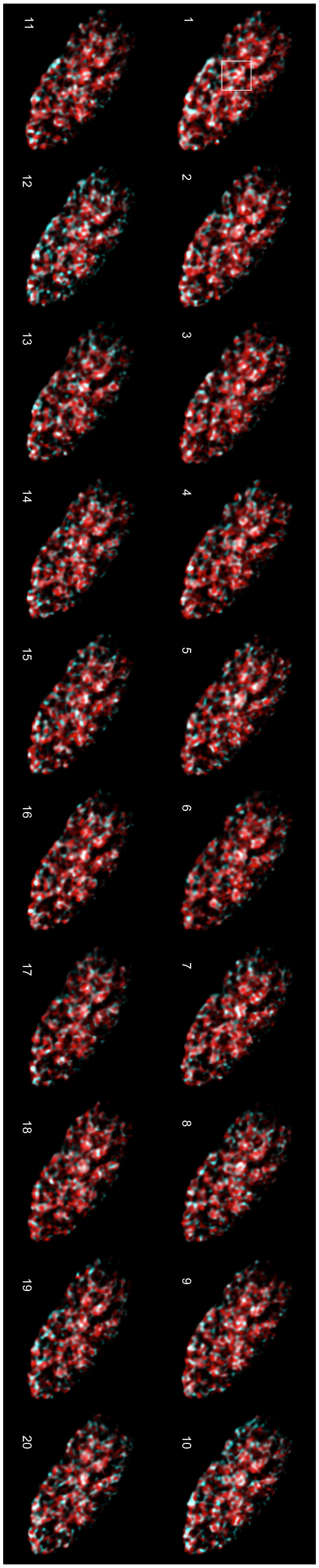

Supplementary Movie 1 | Live-cell imaging of entire chloroplasts in *P. patens* protonema cell. 3D images of WT *P. patens* chloroplasts observed using SCLIM. The image before and after 3D deconvolution is shown in gray and red, respectively. The 3D image was reconstructed using 100 optical sections (x, y) sequentially acquired at 0.05 μm intervals (z = 5 μm), observing Chl fluorescence. Scale units (1 unit = 1.51 μm) and orientation (xyz) are also shown.

Supplementary Movie 2 | Chl in thylakoid membranes and GFP in the stroma spatially excluded from each other. A live cell of *P. patens* line in which GFP is overexpressed in the stroma observed using SCLIM. As shown in Fig. 3, Chl (red) and GFP (green) fluorescence signals are shown. The 3D image was reconstructed using 100 optical sections (x, y) sequentially acquired at 0.05 μm intervals (z = 5 μm), observing Chl and GFP fluorescence. Scale units (1 unit = 0.936 μm) and orientation (xyz) are also shown.

Supplementary Movie 3 | Different structural characteristics observed in two different wavelength regions of Chl fluorescence. A chloroplast in a live WT *P. patens* protonema cell was observed with two different wavelength regions, F_{APM} and F_{PSII}, using SCLIM. As shown in Fig. 5, Chl fluorescence signals for F_{APM} (cyan) and F_{PSII} (red) are shown. The 3D image was reconstructed using 80 optical sections (x, y) sequentially acquired at 0.05 μm intervals (z = 4 μm). In the left panel, scale units (1 unit = 0.714 μm) and orientation (xyz) are shown. In the right panel, the xz slices are shown by moving the different slice positions corresponding to those shown in the left panel. Scale bar in the right panel = 1 μm.

Supplementary Movie 4 | Excitation energy dynamics in a granum structure observed by 3D time-lapse imaging. A chloroplast in a live WT *P. patens* protonema cell observed using SCLIM. As shown in Fig. 6, Chl fluorescence signals for F_{APM} (cyan), F_{PSII} (red), and their merged images are shown. The 3D image was reconstructed using 10 optical sections (x, y) sequentially acquired at 0.05 μm intervals (z = 0.5 μm). The 3D image was sequentially observed with a time interval of 1.47 s. The granum structure is indicated by a white circle.
